# Effects of Probiotics as Adjunctive Therapy to Fluoxetine on Depression Severity and Serum Brain‐Derived Neurotrophic Factor, Cortisol, and Adrenocorticotropic Hormone in Patients With Major Depressive Disorder: A Randomized, Double‐Blind, Placebo‐Controlled Trial

**DOI:** 10.1002/fsn3.4698

**Published:** 2025-04-01

**Authors:** Vajihe Elahinejad, Atie Sadat Khorasanian, Mehdi Tehrani‐Doost, Kianoush Khosravi‐Darani, Zahra Mirsepasi, Mohammad Effatpanah, Reza Askari‐Rabori, Shirin Tajadod, Shima Jazayeri

**Affiliations:** ^1^ Department of Nutrition, School of Public Health Iran University of Medical Sciences Tehran Iran; ^2^ Endocrinology and Metabolism Research Center Endocrinology and Metabolism Clinical Sciences Institute, Tehran University of Medical Sciences Tehran Iran; ^3^ Department of Psychiatry Roozbeh Hospital, Tehran University of Medical Sciences Tehran Iran; ^4^ Research Department of Food Technology Research National Nutrition and Food Technology Research Institute, Shahid Beheshti University of Medical Sciences Tehran Iran; ^5^ School of Medicine Ziaeian Hospital, International Campus, Tehran University of Medical Sciences Tehran Iran; ^6^ Department of Psychiatry Tehran University of Medical Science Tehran Iran; ^7^ Research Center for Nutritional Sciences Iran University of Medical Sciences Tehran Iran

**Keywords:** ACTH, brain‐derived neurotrophic factor, cortisol, depression, major depressive disorder, probiotic

## Abstract

Probiotics may improve mood, but their role as adjunctive therapy for major depressive disorder (MDD) is not well understood. This study examines the effects of probiotics on depression severity, brain‐derived neurotrophic factor (BDNF), adrenocorticotropic hormone (ACTH), and cortisol levels in MDD patients. Fifty medication‐free MDD patients were randomized to receive probiotics with fluoxetine (*n* = 25) or placebo with fluoxetine (*n* = 25) for 8 weeks. Depression severity was assessed using the Hamilton Depression Rating Scale (HDRS‐24), and fasting blood samples were collected at baseline and study conclusion. Forty‐four patients completed the trial. The probiotic group showed a significant reduction in depression severity compared with the placebo group (*p* = 0.001). No significant differences were observed in serum cortisol (*p* = 0.46) and ACTH levels (*p* = 0.44). Plasma BDNF levels increased slightly in the probiotic group but were not statistically significant. Probiotic supplementation with fluoxetine significantly reduces depression severity in MDD patients.

## Graphical abstract

This study evaluates the impact of probiotics on depression severity and biomarkers in MDD patients. Fifty medication‐free MDD patients were randomized to receive probiotics with fluoxetine or placebo with fluoxetine for 8 weeks. Results indicated a significant reduction in depression severity in the probiotic group, though no significant differences were found in cortisol, ACTH, or BDNF levels.

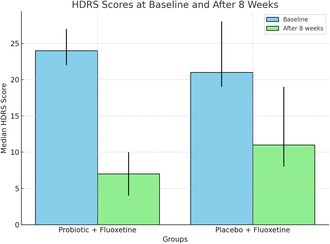

## 1 Introduction

Major depressive disorder (MDD) is a recurring and debilitating condition that poses a significant risk to individuals' lives (Sadock and Sadock 2011; Lakhan and Vieira 2008). Symptoms of depression can impact various aspects of a person's life, including their thoughts, behaviors, emotions, and overall well‐being. MDD is the fourth highest cause of the global disease burden, impacting millions globally. Only a third of those affected respond favorably to conventional antidepressant therapies (Lim et al. 2018; Brenjian et al. 2020). The World Health Organization (WHO) reports that more than 300 million people worldwide are impacted by MDD (Depression 2017).

Given the limitations of current treatments, new therapeutic approaches for MDD are urgently needed. New studies have emphasized the two‐way communication linking the gut and the brain via different routes such as the immune system, neuroendocrine pathways, and the vagus nerve (Cryan and Dinan 2012; Li et al. 2018; Liang et al. 2018). Recent studies increasingly suggest that this connection extends to the intestinal microbiota, triggering the immune system and releasing signaling molecules capable of influencing brain function and subsequent behavior. For instance, the microbiota generates compounds that activate neuromuscular functions and their building blocks, such as tryptophan, which can access the brain via pathways that engage the endocrine and immune systems (Steenbergen et al. 2015).

In both animal studies and in limited research involving humans, there is evidence suggesting that probiotics may have potential benefits in alleviating depression and anxiety (Akkasheh et al. 2016; Desbonnet et al. 2010; Messaoudi et al. 2011; Kazemi et al. 2019; Rudzki et al. 2019). Results from these investigations suggest that probiotics may achieve these impacts via diverse mechanisms, including the inhibition of the hypothalamic–pituitary–adrenal (HPA) axis (Ait‐Belgnaoui et al. 2014), the enhancement of gamma‐aminobutyric acid (GABA) biosynthesis (Dhakal, Bajpai, and Baek 2012), and the promotion of serotonin levels by increasing the production of tryptophan, a precursor to serotonin (Desbonnet et al. 2008). Additionally, neurotrophin factors, such as BDNF, which are crucial for neuronal growth, survival, synaptic function, and neuroplasticity, seem to play a significant role (Huang and Reichardt 2001; Shimizu et al. 2003). Studies indicate that depressed individuals have lower levels of BDNF in their serum or plasma compared with healthy individuals and that antidepressant treatment can boost these diminished BDNF levels (Lee and Kim 2010). In animal research, the intake of probiotics containing *Bifidobacterium longum* R0175 has been linked to enhancements in behavior and the expression of BDNF mRNA in the hippocampus. This outcome is probably due to a rise in short‐chain fatty acids, which function as inhibitors of histone deacetylases (Bercik et al. 2010; Dinan et al. 2015). Interestingly, BDNF levels have been found to be linked with patients' responses to antidepressants rather than the seriousness of their depression, indicating that BDNF might be essential for an antidepressant effect (Björkholm and Monteggia 2016; Wolkowitz et al. 2011). The number of human studies examining the effect of probiotics on MDD is limited. To date, seven studies have investigated this effect in patients with MDD, but inconsistent results have been reported, likely due to variations in bacterial strains and species, intervention duration, failure to control for antidepressants as confounders, dosage, and participant characteristics. Moreover, in previous studies examining the effects of probiotics in patients with MDD, participants either had a history of taking antidepressants or were not taking any antidepressants during the intervention period. Additionally, although medication remained unchanged during the intervention period in some studies, the specific type of antidepressant medication used in combination with probiotics was not controlled (Akkasheh et al. 2016; Kazemi et al. 2019; Rudzki et al. 2019; Majeed et al. 2018; Tian et al. 2022; Wallace and Milev 2021; Chen et al. 2021). A recent meta‐analysis and systematic review indicated that probiotics, when compared to antidepressants and placebos, may be effective as either an adjunct or standalone therapy for treating MDD (Zhao et al. 2024).

This study aimed to build on previous research by investigating the effects of probiotics, specifically *Lactobacillus helveticus* R0052 and *Bifidobacterium longum* R0175, on depression severity and associated mechanisms (e.g., BDNF levels and the HPA axis) in patients diagnosed with MDD who were also receiving fluoxetine. While similar trials have explored probiotics in combination with antidepressants (Arifdjanova et al. 2021; Ghorbani et al. 2018), this study provides additional insights into the potential mechanisms underlying probiotic effects in patients on fluoxetine, a commonly prescribed selective serotonin reuptake inhibitor (SSRI).

## 2 Methods

### 2.1 Participants

This study recruited 50 participants aged 18 to 65 years diagnosed with major depressive disorder (MDD), based on DSM‐IV‐TR criteria, from Ziaeian Hospital. All participants had a minimum Hamilton Depression Rating Scale (HDRS‐24) score of 15 at baseline. Importantly, participants were experiencing their first episode of MDD, and none had received antidepressant medication for at least 2 months prior to enrolment. This washout period was chosen to ensure that the effects of any prior medications did not influence the outcomes of the intervention. Exclusion criteria included substance abuse, severe chronic physical illness, pregnancy or breastfeeding, and psychosis or manic episodes. Participants were also excluded if they were taking probiotics, antibiotics, or nutritional supplements during the intervention period. To further minimize confounding factors, participants who had received psychotherapy or other psychological interventions within 2 months before the study start were excluded, ensuring that any ongoing effects from previous psychological treatments would not impact the results.

### 2.2 Study Design

This study followed a randomized, double‐blind, placebo‐controlled design. Before enrollment, participants were asked about their consumption of any other probiotic or synbiotic foods within 2 weeks. They were then randomly assigned to one of two groups: one group received daily 20 mg of fluoxetine along with either a probiotic or placebo capsule for 8 weeks. Throughout the intervention, participants were instructed to maintain their usual physical activity and diet and to avoid other probiotic or symbiotic products.

### 2.3 Randomization and Blinding

We employed block randomization with a block size of 4 to assign participants to control and intervention groups in balanced blocks, ensuring equal numbers in each group throughout the study. Randomization was conducted using https://www.sealedenvelope.com/ to generate the sequence. To maintain blinding, the probiotic and placebo capsules were identical in appearance, and allocation codes were generated by an independent third party uninvolved in the study. Both researchers and participants remained blinded to group assignments, unaware of whether participants received the placebo or probiotic capsules.

### 2.4 Laboratory Analyses

Ten‐milliliter fasting blood samples were collected at the beginning and after 8 weeks at a medical laboratory in the early morning following an overnight fast. The samples were promptly centrifuged at 3500 rpm for 10 min using a Universal centrifuge from Germany to separate the serum. After centrifugation, the samples were stored at −80°C until analysis at the Noor Medical Laboratory. Plasma BDNF concentration was measured using commercially available enzyme‐linked immunosorbent assay (ELISA) kits sourced from Sigma Aldrich Company US, boasting a sensitivity of 7.8 pg/mL. Serum cortisol concentration was determined utilizing the Chemiluminescence method and commercial kits from DiaSorine s.p.a company, USA, which offered a sensitivity of 1 pg/mL. Furthermore, plasma adrenocorticotropic hormone (ACTH) levels were evaluated using the electrochemiluminescence method and commercial kits from Roche Diagnostics, Germany, featuring a sensitivity of 0.15 μg/dL.

### 2.5 Dietary and Anthropometric Measurements

To mitigate potential confounding factors, dietary intake and physical activity were evaluated before and after the intervention. The participants' nutrient intake was assessed using customized Nutritionist IV software (First Databank, San Bruno, CA, USA) for Iranian foods. Physical activity levels were quantified in metabolic equivalents (METs) per hour per day. METs for each participant were calculated by multiplying the reported duration (hours per day) of each physical activity by its corresponding METs coefficient. On assessment days, participants were instructed to wake up early between 6:30 and 7:00 a.m. and arrive at the laboratory before 8:00 a.m. to standardize the cortisol awakening response. Weight measurements were taken at baseline, 4 weeks, and 8 weeks after the intervention, while participants were fasting overnight, barefoot, and wearing minimal clothing. A digital scale (Omron, Hamburg, Germany) accurate to the nearest 0.1 kg was used for measurements. Height was measured using a nonstretched tape measure (Seca, Hamburg, Germany) accurate to the nearest 0.1 cm. Body mass index (BMI) was then calculated by dividing weight in kilograms by height in meters squared.

### 2.6 Intervention

All participants were administered 20 mg of fluoxetine daily, with a gradual introduction to minimize potential side effects. Specifically, participants started on a reduced dose of 10 mg every 2 days for the first 10 days before transitioning to the full 20 mg daily dose. This approach aligns with clinical guidelines that recommend gradual dose titration to improve tolerability, especially in patients starting antidepressant treatment (Horowitz and Taylor 2024; Cleare et al. 2015; Stahl 2021). The probiotic capsules contained *Lactobacillus helveticus* R0052 (3 × 10^9^ CFU/g) and *Bifidobacterium longum* R0175 (3 × 10^9^ CFU/g), with the dosage determined based on findings from previous study (Messaoudi et al. 2011). Placebo capsules, manufactured by the Department of Pharmacokinetics, School of Pharmacy, Islamic Azad University, Tehran, Iran, consisted of starch without bacteria, identical in appearance to the probiotic capsules. Probiotic capsules were supplied by Takgen Pharma Company, Tehran, Iran, and approved by the Food and Drug Administration of the Islamic Republic of Iran.

### 2.7 Compliance

Compliance with the intervention was assessed by counting the number of capsules returned at follow‐up visit. Participants were provided with a pill diary and instructed to document their daily intake of the probiotic or placebo capsules. Study staff reviewed these diaries and the returned capsules to estimate adherence rates. Compliance was defined as taking at least 80% of the prescribed capsules over the 8‐week study period. The overall compliance rate for both groups was high, with an average of 90% adherence in the probiotic + fluoxetine group and 88% adherence in the placebo + fluoxetine group. No significant differences in compliance rates were observed between the groups. Participants who were found to have lower compliance (< 80%) were provided with reminders and additional guidance to encourage consistent intake. However, compliance issues did not appear to be a significant factor contributing to the dropout rates in the study.

Throughout the intervention period, participants were instructed to refrain from initiating any new psychotherapy sessions. Compliance was assessed at each follow‐up visit by asking participants about any psychological treatments they may have started during the study period.

### 2.8 Outcomes

In this study, the primary outcome measure was depression severity, evaluated using the 24‐item Hamilton Depression Rating Scale (24‐HDRS) at the beginning of the study and after 8 weeks. Secondary outcome measures included levels of BDNF, cortisol, and ACTH.

### 2.9 Adverse Events Collection and Reporting

Adverse events were monitored throughout the study via weekly telephone interviews. Participants were instructed to report any symptoms, side effects, or health changes they experienced during the intervention. All reported events were recorded and assessed for severity and potential relation to the probiotic or placebo. Additionally, clinical staff were available to respond to participant concerns regarding adverse events at any point during the study.

### 2.10 Sample Size

The power calculation for this study was performed based on serum cortisol levels, which was a secondary outcome. This decision was made because there were limited available data on the expected effect size of probiotics on the primary outcome (depression severity), particularly when combined with antidepressants. In contrast, prior studies had reported standard deviations for cortisol levels in similar populations, making it feasible to calculate the required sample size with more confidence. A sample size calculation was performed for serum cortisol levels, with the assumption of at least 80% power (*β* = 0.20) and a significance level (*α*) of 0.05. This calculation was based on a previous study that reported a standard deviation of 2.5 μg/dL for cortisol (Mohammadi et al. 2016). A clinically meaningful effect size of 0.80 (large effect size) was chosen based on the expected difference in cortisol levels between the probiotic and placebo groups. Using these parameters, it was determined that at least 20 participants per group would be required to detect a significant difference. To account for potential dropout, 50 participants were recruited in total.

### 2.11 Statistical Analysis

Data analysis was carried out using SPSS version 16. The normality of the data was assessed using the Kolmogorov–Smirnov test. Depending on the data distribution, the *t*‐test, Mann–Whitney test, and chi‐squared tests were utilized to compare differences between the two groups. At the beginning of the study, a comparison between the two groups was made using the chi‐squared test for categorical variables, and *t*‐tests and Mann–Whitney tests for continuous variables. Nonparametric Wilcoxon tests were used to compare sample parameters within groups. Cortisol, ACTH, and BDNF levels were analyzed using the Mann–Whitney test. A *p* value < 0.05 was considered statistically significant.

## 3 Results

**FIGURE 1:**
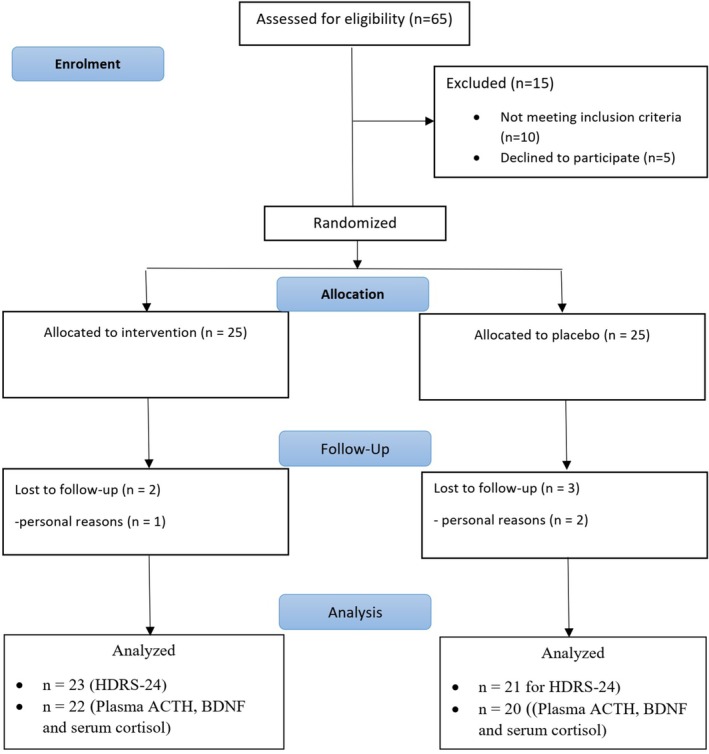
CONSORT diagram.

As shown in Figure 1, out of the initial 65 participants assessed for eligibility, 50 were randomized into two groups: 25 participants in the probiotic + fluoxetine group and 25 in the placebo + fluoxetine group. During follow‐up, two participants in the probiotic group and four in the placebo group were lost to follow‐up, primarily due to unwillingness to cooperate and one case of headache in the placebo group. Ultimately, 23 participants in the probiotic group and 21 in the placebo group completed the study.

Table 1 displays the baseline characteristics of the patients, showing no statistically significant differences between the groups in these aspects. Table 2 presents the dietary intakes of the studied groups, indicating that there were no statistically significant differences between the probiotic + fluoxetine group and the placebo + fluoxetine group across various dietary components.

**TABLE 1:** Baseline characteristics of participants.

	Probiotic+ fluoxetine (*n* = 23)	Placebo+ fluoxetine (*n* = 21)	*p*
Age (years)^a^, ^d^	36.80 ± 9.64	34.68 ± 9.14	0.71
Weight (Kg)^a^	75.58 ± 12.26	76.58 ± 13.59	0.40
BMI (Kg/m^2^)^a^, ^e^	28.70 ± 4.96	28.79 ± 4.93	0.47
Gender, *n* (%)^b^			
Male	3 (13)	2 (9.5)	0.54
Female	20 (87)	19 (90.5)	
Hypertension, *n* (%)^b^			
Yes	2 (8.7)	1 (4.8)	0.53
No	21 (91.3)	20 (95.2)	
Smoking, *n* (%)^b^			
Yes	3 (13)	2 (9.5)	0.54
No	20 (87)	19 (90.5)	
Family history of depression, *n* (%)^b^			
Yes	11 (47.8)	8 (38.1)	0.36
No	12 (52.2)	13 (61.9)	
SBP (mmg)^a^, ^f^	105.21 ± 13.43	99.25 ± 13.20	0.50
DBP (mmg)^a^, ^f^	70.86 ± 10.83	63.00 ± 10.80	0.98
Age of onset (years)^a^, ^g^	27.22 ± 7.83	27.50 ± 7.81	0.86
Symptom severity at baseline (HDRS score)^c^	24 (22–27)	21 (19–28)	0.22
Physical activity (METs)^c^	693 (404.25–1313)	990 (272–4156)	0.41

Abbreviations: BMI, body mass index; DBP, diastolic blood pressure; HDRS, Hamilton Depression Rating Scale; METs, metabolic equivalent of tasks; SBP, systolic blood pressure.

^a^ The variable has a normal distribution, and the values are reported as mean ± standard deviation. The *p* value between groups was calculated based on the independent *t*‐test.

^b^ Using Fisher's exact test, the values are reported as frequency and percentage.

^c^ The variable has a non‐normal distribution, and the values are reported as median and interquartile range (25th–75th percentile). The *p* value between groups was calculated based on the Mann–Whitney test.

^d^ As to the age variable analysis, there are 21 patients in the intervention group and 19 patients in the placebo group.

^e^ As to the BMI variable analysis, there are 22 patients in the intervention group and 21 patients in the placebo group.

^f^ As to the SBP and DBP variable analysis, there are 23 patients in the intervention group and 20 patients in the placebo group.

^g^ As to the age of onset variable analysis, there are 18 patients in the intervention group and 18 patients in the placebo group.

**TABLE 2:** Dietary intakes in the studied groups.

	Probiotic+ fluoxetine (*n* = 23)	Placebo+ fluoxetine (*n* = 21)	*p*
Energy (kcal/d)^a^	1695.01 ± 608.32	1702.62 ± 665.61	0.90
Carbohydrates (g/d)^b^	205.5 (142.4–270.3)	197.3 (154.2–269.6)	0.41
Fat (g/d)^a^	67.70 ± 37.40	76.11 ± 38.45	0.40
Protein (g/d)^a^	55.34 ± 26.80	56.08 ± 25.28	0.75
Cholesterol (mg/d)^b^	115.55 (64.44–232.27)	119.73 (70.26–171.85)	0.40
Fiber (g/d)^b^	7.03 (3.48–15.07)	9.28 (5.26–13.06)	0.96
Vitamin A (μg/d)^b^	293.8 (164.11–415.9)	224.80 (67.88–720.75)	0.89
Vitamin D (μg/d)^b^	0 (0–0.38)	0 (0–0.21)	0.66
Vitamin B1 (mg/d)^a^	1.64 ± 0.59	1.58 ± 0.65	0.94
Vitamin B12 (μg/d)^b^	1.72 (0.66–2.51)	1.36 (0.78–2.46)	0.78
Folic acid (μg/d)^b^	149.35 (78.56–223.15)	111 (75.36–169.35)	0.22
Vitamin C (mg/d)^b^	24.49 (13.77–7512)	39.06 (11.08–86.46)	0.81
Calcium (mg/d)^a^	556.07 ± 275.93	509.62 ± 315.60	0.66
Iron (mg/d)^b^	12.33 (8.79–18.03)	13.95 (9.99–16.09)	0.77
Magnesium (mg/d)^a^	243.29 ± 117.06	222.81 ± 106.13	0.67
Zinc (mg/d)^b^	7.67 (3.78–9.68)	7.75 (4.95–9.24)	0.91
Selenium (μg/d)^a^	0.08 ± 0.05	0.08 ± 0.05	0.96

^a^ The variable has a normal distribution, and the values are reported as mean ± standard deviation. The *p* value between groups was calculated based on the independent *t*‐test.

^b^ The variable has a non‐normal distribution, and the values are reported as median and interquartile range (25th–75th percentile). The *p* value between groups was calculated based on the Mann–Whitney test.

**FIGURE 2:**
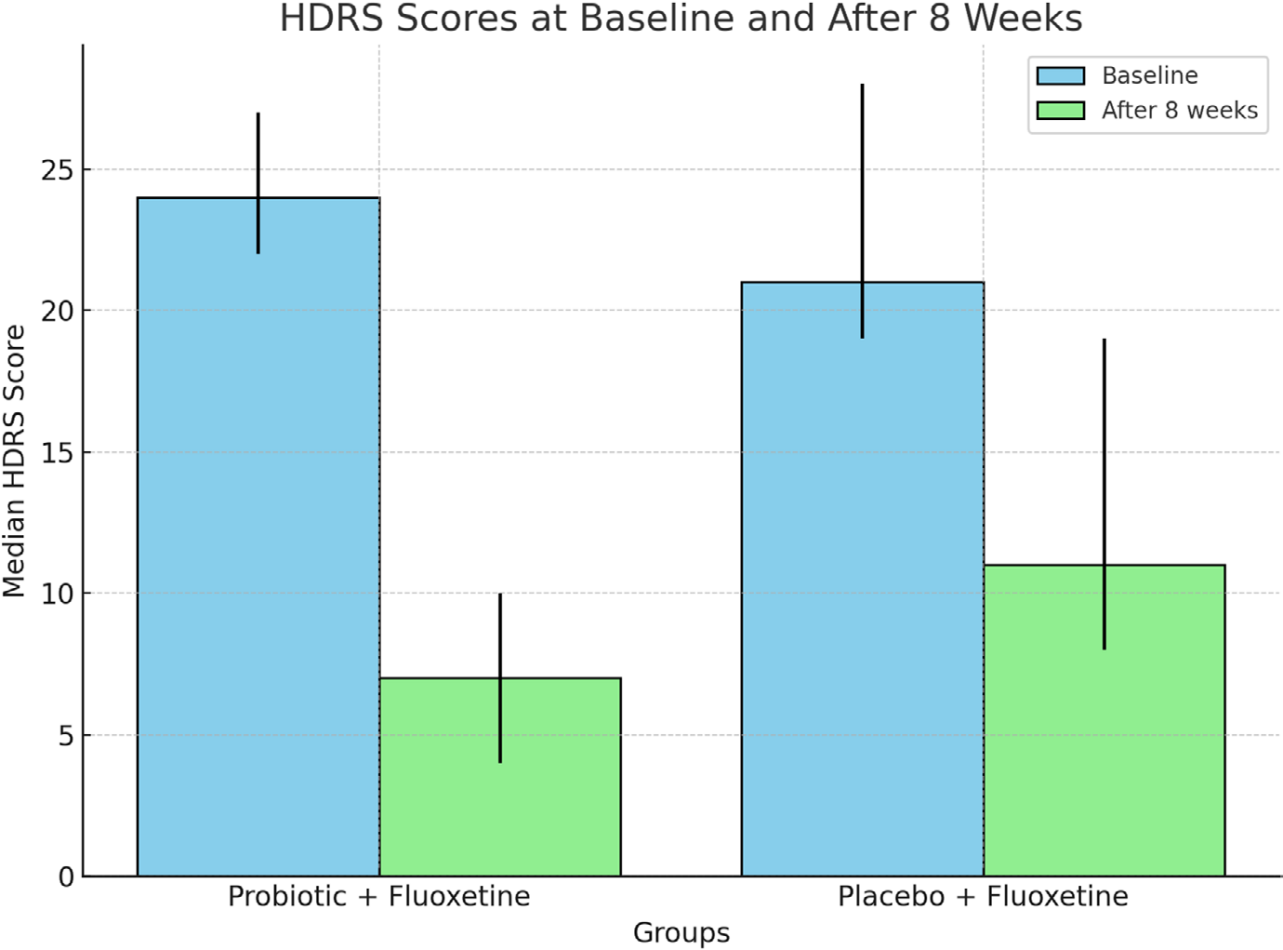
Median Hamilton Depression Rating Scale (HDRS‐24) scores at baseline and after 8 weeks for Probiotic + Fluoxetine (*n* = 23; baseline IQR: 22–27, 8‐week IQR: 4–10) and Placebo + Fluoxetine (*n* = 21; baseline IQR: 19–28, 8‐week IQR: 8–19) groups. Both groups showed significant reductions in scores after 8 weeks (*p* < 0.001 within groups), with a greater reduction observed in the Probiotic + Fluoxetine group at 8 weeks (*p* < 0.001).

As shown in Table 3, there was a significant reduction in HDRS‐24 scores in both groups from baseline to 8 weeks. The probiotic + fluoxetine group showed a median decrease in HDRS‐24 score from 24 (IQR: 22–27) to 7 (IQR: 4–10), while the placebo + fluoxetine group decreased from 21 (IQR: 19–28) to 11 (IQR: 8–19), both with *p* < 0.001. However, after 8 weeks, the probiotic + fluoxetine group demonstrated a significantly greater improvement compared with the placebo group (*p* < 0.001). Also, Figure 2 illustrates median HDRS‐24 scores at baseline and after 8 weeks for both groups. Table 4 provides detailed information on plasma ACTH, BDNF, and serum cortisol levels for both the probiotic + fluoxetine group and the placebo + fluoxetine group, measured at baseline and after 8 weeks of intervention. The data indicate that there were no significant changes in any of these biomarkers within each group over the 8‐week period, nor were there any statistically significant differences between the two groups at either time point (*p* > 0.05).

**TABLE 3:** Hamilton Depression Rating Scale (HDRS‐24) at baseline and after 8 weeks.

	Groups/*p*	Baseline	After 8 weeks	*p*
HDRS	Probiotic + fluoxetine (*n* = 23)	24 (22–27)	7 (4–10)	0.001
	Placebo + fluoxetine (*n* = 21)	21 (19–28)	11 (8–19)	0.001
	*p*	0.22	0.001	

*Note:* The variable has a non‐normal distribution, and the values are reported as median and interquartile range (25th–75th percentile). The *p* value within each group was calculated based on the Wilcoxon test.

**TABLE 4:** Plasma ACTH, BDNF and serum cortisol at baseline and after 8 weeks.

	Probiotic+ fluoxetine (*n* = 22)	Placebo+ fluoxetine (*n* = 20)	*p*
Cortisol (μg/dL)			
Baseline	13.85 (11.47–18.77)	15.70 (10.42–18.00)	0.75
Week 8	13.50 (9.62–19.07)	12.75 (9.40–18.77)	0.98
*p*	0.63	0.86	
ACTH (pg/mL)			
Baseline	25.95 (17.15–37.12)	21.60 (16.35–33.02)	0.46
Week 8	20.75 (12.27–32.67)	19.45 (14.40–55.10)	0.68
*p*	0.98	0.28	
BDNF (pg/mL)			
Baseline	68 (45.5–108.5)	62 (38–112.5)	0.62
Week 8	76 (50.5–108.5)	52 (38.5–135.5)	0.71
*p*	0.55	0.89	

*Note:* The variables have a non‐normal distribution, and the values are reported as median and interquartile range (25th–75th percentile). The *p* value within each group was calculated based on the Wilcoxon test.

Abbreviations: ACTH, adrenocorticotropic hormone; BDNF, brain‐derived neurotrophic factor.

### 3.1 Adverse Events

No serious adverse events were reported during the course of the study. Minor adverse events were reported by participants in the probiotic group, including mild gastrointestinal symptoms such as bloating, gas, and abdominal discomfort, which were transient and resolved without the need for medical intervention. Additionally, one participant in the placebo + fluoxetine group reported a persistent headache, which led to their withdrawal from the study.

## 4 Discussion

This study demonstrated that an 8‐week probiotic supplementation alongside fluoxetine led to a positive impact on depression severity in patients with MDD. However, there was no significant influence on HPA axis indicators or plasma BDNF levels. Although BDNF levels increased in the probiotic group, these changes did not reach statistical significance.

Our study found that 8 weeks of probiotic supplementation combined with fluoxetine significantly reduced depression severity in patients with MDD. Nikolova et al. (2023) reported substantial reductions in both depressive and anxiety symptoms with probiotic use alongside antidepressants, emphasizing high tolerability and adherence to probiotics in combination therapy (Nikolova et al. 2023). Similarly, Schaub et al. (2022) observed decreased depressive symptoms along with increased Lactobacillus levels, suggesting that microbiota modulation through probiotics may influence mood via the gut–brain axis (Schaub et al. 2022). Studies on synbiotics, which combine probiotics with prebiotics, also support probiotics' effectiveness in depression management. Ghorbani et al. (2018) demonstrated that synbiotics combined with fluoxetine significantly reduced depression severity, further supporting the gut–brain axis as a potential mechanism in depression treatment (Ghorbani et al. 2018). Arifdjanova et al. (2021) reported similar findings, with Bac‐Set Forte (a 14‐strain probiotic) in combination with escitalopram showing significant improvements in both depressive symptoms and neuroimmune endocrine markers, reinforcing the impact of probiotics on the gut–brain axis and inflammatory pathways in depressive disorders (Arifdjanova et al. 2021). While our study did not investigate prebiotics, our inclusion of biomarkers such as BDNF and cortisol provides additional insights into potential mechanisms through which probiotics might influence mood. Despite observing an increase in BDNF levels in the probiotic group, the changes did not reach statistical significance, possibly due to the relatively short intervention duration or the normal baseline values in our participants. Similarly, cortisol and ACTH levels remained unchanged, which may suggest that probiotics require longer‐term administration to exert measurable effects on the HPA axis. These findings highlight the need for further studies with extended intervention periods and larger sample sizes to better elucidate these mechanisms.

Further supporting these findings, several studies indicate that probiotics positively impact depression severity. For example, Kazemi et al. and Akkasheh et al. both reported significant reductions in depression severity after 8 weeks of probiotic intervention. Kazemi et al. used the same probiotic strain as our study in participants who had been on antidepressants for at least 3 months, while Akkasheh's study involved patients who were not on any medication (Akkasheh et al. 2016; Kazemi et al. 2019). Additionally, Majeed et al. (2018) demonstrated improvements in both depression and irritable bowel syndrome (IBS) symptoms with *Bacillus coagulans* MTCC 5856 (Majeed et al. 2018). Wallace and Milev reported notable symptom improvements in treatment‐naïve MDD patients using probiotics alone (Wallace and Milev 2021), while Chen et al. observed a decrease in depression severity with *Lactobacillus plantarum* PS128 (Chen et al. 2021). Tian et al. (2022) also found that *Bifidobacterium breve* CCFM1025 significantly reduced depression severity after 4 weeks in patients not taking antidepressants (Tian et al. 2022). Probiotics may help alleviate depression by influencing the gut–brain axis. They can potentially synthesize neurotransmitters such as GABA, produced by Lactobacillus and Bifidobacteria, and serotonin, produced by Candida and *Escherichia coli* (Goh et al. 2019; Fond et al. 2015). Additionally, probiotics may reduce inflammation and help regulate gut microbiota (Cepeda, Katz, and Blacketer 2017).

Contrasting these positive findings, some studies report modest effects of probiotics on depressive symptoms and inconsistent impacts on gut microbiota diversity. A recent systematic review found that most studies showed limited changes in gut microbiota composition following probiotic intervention, often with only modest improvements in depressive symptoms (Ng et al. 2023). Similarly, Rudzki et al. found no relationship between *Lactobacillus plantarum* 299v and depression severity, likely due to its ineffectiveness in altering plasma tryptophan levels, a key factor in serotonin synthesis (Rudzki et al. 2019). Romijn et al. also reported no significant symptom improvements in participants who were not using psychotropic medications, suggesting that probiotic efficacy may depend on concurrent medication use or treatment regimens (Romijn et al. 2017). Variations in formulations and biomarkers among studies may explain the differing outcomes observed.

This study uniquely examines the effects of probiotics on plasma BDNF levels in individuals with MDD who were medication‐free for at least 2 months prior to the intervention. Previous research has primarily focused on populations concurrently using antidepressants or other medications, leaving a gap in understanding how probiotics influence BDNF levels independently of pharmaceutical effects (Huang, Wang, and Hu 2016; Noonan et al. 2020; Ng et al. 2018). Neurotrophic factors like BDNF are crucial for preventing cellular atrophy and neuronal loss in depression, and individuals with depression typically show lower BDNF levels (Jones and Reichardt 1990; Banasr, Dwyer, and Duman 2011). While animal studies suggest that probiotics can elevate BDNF levels in the hippocampus, there is limited evidence in humans (Ait‐Belgnaoui et al. 2014; Tian et al. 2019; Liang et al. 2015). Recent randomized controlled trials have generally shown no significant impact of probiotics on plasma BDNF levels in both healthy individuals and those with low mood (Romijn et al. 2017; Chung et al. 2014). Notably, a study by Haghighat et al. found that while probiotics (*Lactobacillus acidophilus T16, Bifidobacterium bifidum BIA‐6, Bifidobacterium lactis BIA‐7*, and *Bifidobacterium longum BIA‐8*) did not significantly improve serum BDNF, a synbiotic treatment did, especially in depressed patients.

(Haghighat, Rajabi, and Mohammadshahi 2021).

Two clinical trials examining probiotic supplementation (one with *Lactobacillus helveticus* R0052 and *Bifidobacterium longum* R0175 for 8 weeks, and the other with *Bifidobacterium longum* R0175 for 6 weeks) showed no significant impact on plasma BDNF levels in individuals with low mood and IBS (Romijn et al. 2017; Pinto‐Sanchez et al. 2017). Most studies on BDNF in depressed individuals had longer intervention durations, typically around 12 weeks (Solati et al. 2015; Yeh et al. 2015). In contrast, Heidarzadeh‐Rad et al. found that an 8‐week supplementation of probiotics with *Lactobacillus helveticus* R0052 and *Bifidobacterium longum* R0175 significantly increased serum BDNF levels in patients with mild to moderate melancholic depression (Heidarzadeh‐Rad et al. 2020). In our study, although plasma BDNF levels showed a slight increase in the probiotic group, this change did not reach statistical significance. This may be attributed to the short duration of the intervention or the relatively normal baseline BDNF levels in the study population. Additionally, the lack of significant findings could indicate that probiotics exert their influence on mood through mechanisms other than directly altering peripheral BDNF levels. Further studies with longer intervention durations and broader participant demographics are needed to clarify these effects.

Probiotic supplementation for 8 weeks did not alter HPA axis indicators, such as serum cortisol and plasma ACTH, in patients with MDD. Research on this topic is limited, though some animal studies suggest that probiotics can regulate HPA axis activity and cortisol levels (Ait‐Belgnaoui et al. 2014; Gareau et al. 2007). Consistent with our findings, a systematic review and meta‐analysis on probiotics and stress, as well as a study by Leszek Rudzki et al., reported no significant differences in serum cortisol levels between intervention and placebo groups over 8 weeks (Rudzki et al. 2019; Zhang et al. 2020). The absence of significant changes in cortisol and ACTH levels in our study could be attributed to the relatively normal baseline values of these markers in the participants, reducing the likelihood of detecting measurable changes. Additionally, the intervention duration may have been insufficient for probiotics to influence the HPA axis meaningfully.

A 6‐week administration of probiotic yogurt or capsules to petrochemical workers did not significantly affect serum ACTH levels (Mohammadi et al. 2016). In contrast, Romijn et al. found that a multibiotic combined with escitalopram significantly reduced cortisol levels after 6 weeks in patients with mild to moderate depressive episodes (Romijn et al. 2017). Similarly, Nishihira et al. reported that 12 months of yogurt containing *Lactobacillus gasseri GR1* and *Bifidobacterium longum* R0175 significantly decreased serum ACTH levels in healthy participants (Nishihira et al. 2014). These contrasting findings suggest that the impact of probiotics on the HPA axis may depend on the population studied, baseline cortisol levels, or the specific strains and duration of supplementation.

### 4.1 Strengths and Limitations

This study had notable strengths, including strict control over medication use to minimize confounding factors and the inclusion of biomarkers such as BDNF and cortisol to explore potential neuroendocrine effects. However, limitations included a short intervention period and financial constraints, which restricted long‐term biomarker analysis and prevented assessment of gut microbiota changes. The predominantly female sample limits generalizability, and specific probiotic strains were not analyzed, leaving strain‐specific effects on depression unclear. Future research with longer durations, diverse samples, and strain‐specific analyses is recommended to address these aspects.

## 5 Conclusion

In conclusion, over an 8‐week period, adjunctive therapy with probiotic bacteria *Lactobacillus helveticus* R0052 (3 × 10^9^ CFU/g) and *Bifidobacterium longum* R0175 (3 × 10^9^ CFU/g) demonstrated a reduction in depression severity among patients with MDD. However, this treatment did not have a significant impact on the HPA axis or plasma BDNF. Future research should consider conducting studies with expanded sample sizes and extended durations to delve deeper into the potential mechanisms through which probiotics exert their effects on depression.

## Author Contributions

**Vajihe Elahinejad:** conceptualization (equal), data curation (equal), formal analysis (equal), investigation (equal), methodology (equal), software (equal), validation (equal). **Atie Sadat Khorasanian:** formal analysis (equal), writing – original draft (equal), writing – review and editing (equal). **Mehdi Tehrani‐Doost:** methodology (equal), validation (equal), writing – review and editing (equal). **Kianoush Khosravi‐Darani:** conceptualization (equal), methodology (equal), writing – review and editing (equal). **Zahra Mirsepasi:** conceptualization (equal), methodology (equal), writing – review and editing (equal). **Mohammad Effatpanah:** conceptualization (equal), methodology (equal), writing – review and editing (equal). **Reza Askari‐Rabori:** methodology (equal), writing – original draft (equal), writing – review and editing (equal). **Shirin Tajadod:** investigation (equal), writing – review and editing (equal). **Shima Jazayeri:** conceptualization (equal), data curation (equal), formal analysis (equal), methodology (equal), software (equal), supervision (equal), validation (equal), writing – original draft (equal), writing – review and editing (equal).

## Ethics Statement

The study protocol was approved by the Ethics Committee of Iran University of Medical Sciences, Tehran, Iran (IR.IUMS.REC 1394.26635) and was registered in the Iranian Registry of Clinical Trials (IRCT201601102394N20). All participants provided informed written consent, and the study was conducted in adherence to the principles outlined in the Helsinki Declaration.

## Consent

The authors have nothing to report.

## Conflicts of Interest

The authors declare no conflicts of interest.

## Data Availability

The information supporting the conclusions of this study can be obtained from the corresponding author upon request.
